# *VvMYB14* participates in melatonin-induced proanthocyanidin biosynthesis by upregulating expression of *VvMYBPA1* and *VvMYBPA2* in grape seeds

**DOI:** 10.1093/hr/uhac274

**Published:** 2022-12-07

**Authors:** Xiaoqian Zhang, Wanyun Ma, Xueqiang Guan, Fei Wang, Zongbao Fan, Shiwei Gao, Yuxin Yao

**Affiliations:** State Key Laboratory of Crop Biology, Collaborative Innovation Center of Fruit & Vegetable Quality and Efficient Production, College of Horticulture Science and Engineering, Shandong Agricultural University, Tai-An, Shandong 271018, China; State Key Laboratory of Crop Biology, Collaborative Innovation Center of Fruit & Vegetable Quality and Efficient Production, College of Horticulture Science and Engineering, Shandong Agricultural University, Tai-An, Shandong 271018, China; Shandong Academy of Grape/Shandong Technology Innovation Center of Wine Grape and Wine, Jinan, Shandong 250100, China; State Key Laboratory of Crop Biology, Collaborative Innovation Center of Fruit & Vegetable Quality and Efficient Production, College of Horticulture Science and Engineering, Shandong Agricultural University, Tai-An, Shandong 271018, China; State Key Laboratory of Crop Biology, Collaborative Innovation Center of Fruit & Vegetable Quality and Efficient Production, College of Horticulture Science and Engineering, Shandong Agricultural University, Tai-An, Shandong 271018, China; State Key Laboratory of Crop Biology, Collaborative Innovation Center of Fruit & Vegetable Quality and Efficient Production, College of Horticulture Science and Engineering, Shandong Agricultural University, Tai-An, Shandong 271018, China; State Key Laboratory of Crop Biology, Collaborative Innovation Center of Fruit & Vegetable Quality and Efficient Production, College of Horticulture Science and Engineering, Shandong Agricultural University, Tai-An, Shandong 271018, China

## Abstract

This work demonstrated that melatonin increases continuously in seeds, particularly seed coats, during berry ripening. Exogenous melatonin treatments significantly increased the proanthocyanidin (PA) content, partially through ethylene signaling, in seed coats. *VvMYB14* expression exhibited patterns similar to melatonin accumulation over time, which was largely induced by melatonin treatment in seed coats during berry ripening. Additionally, VvMYB14 bound to the MBS element of the *VvMYBPA1* promoter to activate expression. *VvMYB14* overexpression largely upregulated expression of *VvMYBPA1*, *VvMYBPA2* and *VvLAR1* and increased the PA content in grape seed-derived calli. Similar increases in *AtTT2* and *AtBAN* expression and PA content were found in *VvMYB14*-overexpressing *Arabidopsis* seeds. It was also observed that *VvMYB14* overexpression increased ethylene production and thereby induced expression of *VvERF104*, which bound to the ERF element of the *VvMYBPA2* promoter and activated its expression. Additionally, *VvERF104* suppression reduced the *VvMYB14* overexpression-induced increases in expression of *VvMYBPA2* and *VvLAR1* and PA content. Further experiments revealed that melatonin-induced increases in the expression of *VvMYBPA1*, *VvMYBPA2*, *VvERF104* and *VvLAR1* and PA accumulation were significantly reduced in *VvMYB14*-suppressing grape calli and leaves. Collectively, VvMYB14 mediates melatonin-induced PA biosynthesis by directly transactivating *VvMYBPA1* expression and indirectly upregulating *VvMYBPA2* expression via VvERF104.

## Introduction

Grape is one of the most important fruit crops worldwide. Approximately half of all grapes are used to produce wine, and one-third are consumed as fresh fruit; the remainder is used for other grape products. Grapes are among the richest sources of polyphenols, including proanthocyanidins (PAs). PAs as the second most abundant polyphenolic compounds and are widely present in many plant tissues including bark, leaves, fruits and seeds [1, 2]. Approximately 30% of total PAs are stored in grape seeds and 15% in grape skin [3]. PAs not only greatly affect the astringent or rough sensation of red wine but also may act as strong antioxidants to provide benefits to human health.

Flavan-3-ol monomers, primarily (+)-catechin and (−)-epicatechin, serve as precursors for PA synthesis [1, 2]. Anthocyanidin reductase (ANR) catalyzes the conversion of cyanidin to epicatechin [4]. Two grape leucoanthocyanidin reductases (LARs) convert leucocyanidin to catechin and epicatechin; however, they more efficiently produce catechin [5]. In contrast, the mechanism of flavan-3-ol condensation to produce PA oligomers and polymers remains unknown. Additionally, grapevine VvMYBPA1 and VvMYBPA2 reportedly control expression of genes involved in PA biosynthesis, including *VvLAR1* and *VvANR*, with overexpression of *VvMYBPA1* or *VvMYBPA2* largely increasing the PA content in grapevine hairy roots [6, 7].

Melatonin is an indoleamine that is synthesized from L-tryptophan metabolism via serotonin; it is not only a strong antioxidant [8] but also a multifunctional signaling molecule in plants [9]. It has been reported that melatonin plays a key role in promoting fruit ripening and delaying postharvest senescence in grape, banana and tomato [10–12]. Additionally, melatonin has been reported to increase accumulation of sucrose and sorbitol in pear fruits [13]. Postharvest treatment with melatonin has demonstrated its key role in increasing or maintaining the content of total anthocyanins, total flavonoids, and total phenols in strawberry fruits [14]. In particular, our previous study elucidated that melatonin alters the profile of 27 secondary metabolites in grape berry skin [15]. Therefore, melatonin accelerates fruit ripening and affects metabolite accumulation; however, the pathways involved in melatonin sensing and signaling remain largely unknown. Several studies have shown that melatonin modulates gene expression related to metabolism of ABA, indole-3-acetic acid, cytokinins, gibberellins and ethylene [16], and our previous study revealed that melatonin functions partially through ethylene signaling in regulation of berry ripening [17].

**Figure 1:**
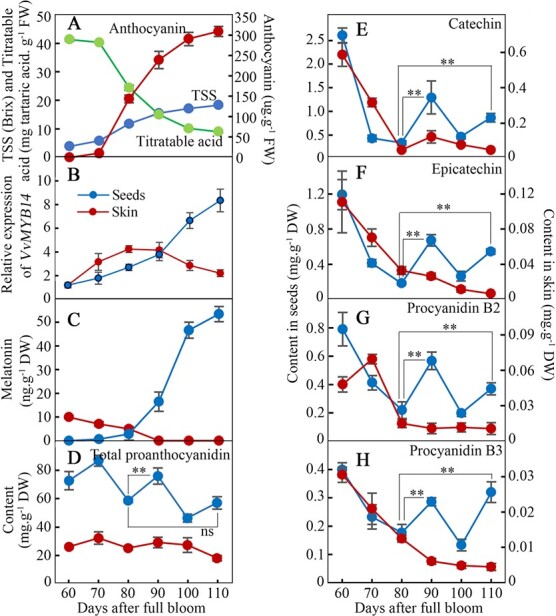
Expression of *VvMYB14* and accumulation of melatonin and PAs during berry ripening. Changes in TSS, titratable acid, and total anthocyanin contents were used to indicate the onset of ripening (A). In Panels B-H, the red and blue lines represent skin and seeds, respectively. Panels E-H share the same coordinate axis titles. The values represent the means ± SD of three replicates. ^*^, Significant difference, P < 0.05; ^**^, highly significant difference, P < 0.01. ns, not significant at P < 0.05.

Additionally, our previous studies revealed that *VvMYB14* might participate in melatonin signaling during secondary metabolite metabolism regulation in Merlot berry skin; the role of *VvMYB14* in regulating accumulation of secondary metabolites was elucidated by its overexpression in Merlot grape berry skin-derived calli [15]. It was also reported that MtMYB14 and MtMYB5 physically interact and synergistically activate expression of *ANR* and *LAR* and that *MtMYB14* overexpression strongly induces PA accumulation in *Medicago truncatula* [18]. Therefore, a pathway by which *VvMYB14* regulates PA accumulation in response to melatonin might exist. Nonetheless, such a pathway might not be a primary pathway involved in PA regulation in berry skin because the melatonin content declines sharply during ripening and is undetectable in the skin at late ripening stages [15]. Although the melatonin accumulation pattern in grape seeds remains unknown, because PAs are stored predominantly in grape seeds [3], we hypothesized that melatonin promotes PA biosynthesis primarily via a *VvMYB14*-mediated pathway in grape seeds.

To test the above hypothesis, accumulation patterns of melatonin, PA and *VvMYB14* transcripts in seeds during berry ripening were determined. The function of melatonin in promoting PA biosynthesis was demonstrated in grape seed coats. The role of *VvMYB14* in regulating PA biosynthesis by modulating expression of *VvMYBPA1* and *VvMYBPA2* or their homologous genes in *Arabidopsis* was revealed through its overexpression in Merlot grape berry seed-derived calli and *Arabidopsis* seeds. Moreover, the role of *VvMYB14* in mediating melatonin-induced PA biosynthesis was determined using *VvMYB14*-suppressed grape calli and grape leaves. This research provides insight into the molecular mechanism underlying melatonin signaling in regulation of PA biosynthesis in grape seeds.

**Figure 2:**
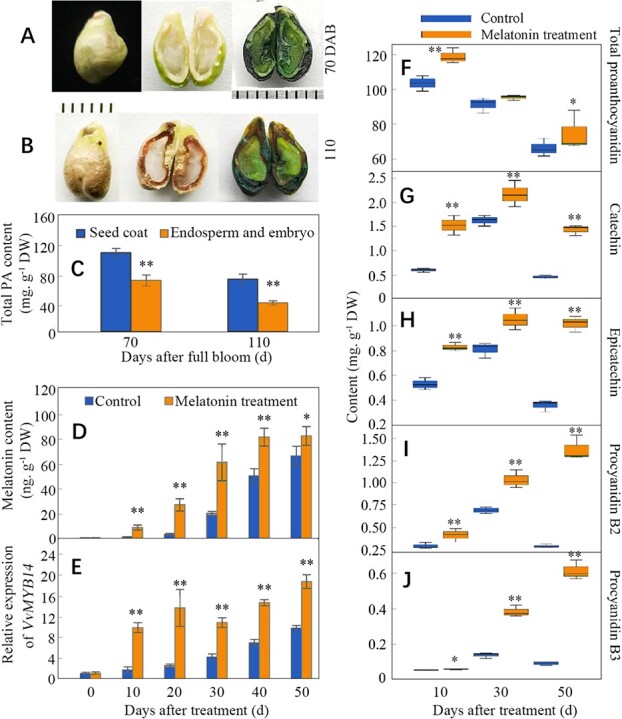
Changes in contents of melatonin and PAs and expression of *VvMYB14* in seed coats under melatonin treatment. Histochemical staining was performed using the seeds from the berries at 70 and 110 DAB (A, B). The scale bar is one millimeter in Panels A and B. The total PA content in different parts of the seeds is shown in Panel C. Melatonin content and *VvMYB14* expression were determined using the seed coats from the berries at different DAB (D, E). The berries were treated at 60 DAB, corresponding to 0 DAT (D-J). Panels F-J share the same y-axis titles. The values represent the means ± SD of three replicates. ^*^, significant difference, P < 0.05; ^**^, highly significant difference, P < 0.01. ns, not significant at P < 0.05.

## Results

### Changes in expression of *VvMYB14* and contents of melatonin and PAs during berry ripening

Total soluble solids (TSS), titratable acid, and total anthocyanin contents were determined to evaluate the berry ripening process. TSS and anthocyanins began to accumulate, and titratable acid began to decrease at 70 days after full bloom (DAB; Fig. 1A), indicating the beginning of berry ripening. Expression of *VvMYB14* continued to increase from 70 DAB and reached a maximum in the seeds of ripened berries; in contrast, expression of this gene decreased in berry skin after 80 DAB (Fig. 1B). The melatonin content showed very similar patterns to those of *VvMYB14* in seeds and was undetectable in the skin during late ripening stages (Fig. 1C). Generally, PA contents in seeds and skin were high before the onset of ripening (Fig. D-H). Compared to values at 80 DAB, when melatonin began to accumulate, the contents of total PAs and four PA compounds (catechin, epicatechin, procyanidin B2 and procyanidin B3) were significantly increased at 90 and 110 DAB in seeds, suggesting the possible positive contribution of melatonin in inducing PA accumulation. However, the low level of PAs at 100 DAB suggested the existence of other negative regulators of PA accumulation. In contrast, the PA content generally showed a continuous decreasing trend in the skin (Fig. D-H).

### Exogenous melatonin treatment increases *VvMYB14* expression and PA accumulation in seed coats

Histochemical staining and content determination showed that PAs primarily accumulated in seed coats and were also detected in the endosperm and embryo at 70 and 110 DAB (Fig. 2A-C). Therefore, seed coats were used to further evaluate the effects of melatonin treatment on *VvMYB14* expression and PA accumulation. Melatonin treatment significantly increased the content of melatonin and the expression level of *VvMYB14* in seed coats on different days after treatment (DAT) (Fig. 2D, E). Additionally, melatonin treatment significantly increased the content of total PAs and four PA compounds at the three time points detected, particularly at 50 DAT (Fig. 2F-J). Overall, increases in procyanidin B2 and procyanidin B3 reached 4.48- and 7.01-fold, respectively, in melatonin-treated seed coats compared with controls at 50 DAT (Fig. 2I, J). Moreover, expression of *VvMYBPA1*, *VvMYBPA2*, *VvLAR1* and *VvLAR2* was upregulated to varying extents at 10, 30 and 50 DAT (Supplementary Data Fig. S1). Melatonin treatment significantly increased the melatonin content and *VvMYB14* expression but significantly reduced the content of PAs, accompanied by reduced expression of *VvMYBPA1*, *VvMYBPA2*, *VvANR*, *VvLAR1* and *VvLAR2,* in berry skin at 10 and/or 30 DAT (Supplementary Data Fig. S2). Therefore, melatonin increased both *VvMYB14* expression and PA accumulation in seed coats but not in berry skin.

### Exogenous melatonin treatment increases PA accumulation partially via ethylene production in seed coats

**Figure 3:**
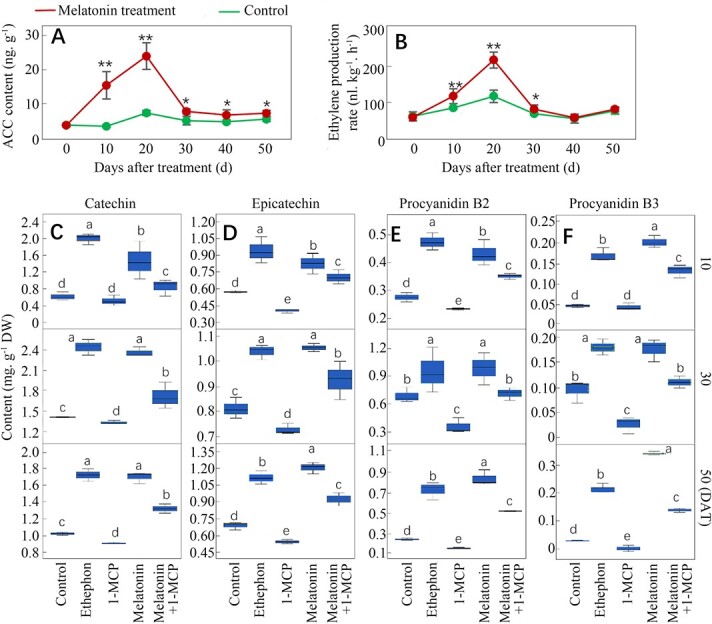
The levels of ethylene production and PA accumulation in the control seed coats and those treated with ethephon, 1-MCP, melatonin, and melatonin plus 1-MCP. The ethylene production level was evaluated by the determination of the ACC (precursor of ethylene) content (A) and ethylene production rate (B). The contents of catechin (C), epicatechin (D), procyanidin B2 (E) and procyanidin B3 (F) were determined in seed coats at 10, 30 and 50 DAT. The berries were treated at 60 DAB, corresponding to 0 DAT. The values represent the mean ± SD of three replicates. ^*^, significant difference, P < 0.05; ^**^, highly significant difference, P < 0.01. The values indicated by the different lowercase letters are significant at P < 0.05.

Our previous study found that exogenous melatonin treatment increased ethylene production in grape berries [17]. In the present, we determined whether melatonin regulates PA synthesis via ethylene production in seed coats. Melatonin treatment significantly enhanced the ACC (precursor of ethylene synthesis) content and ethylene production rate at 10–30 DAT in seed coats, and the largest increases were observed at 20 DAT (Fig. 3A, B). Compared to the control, ethephon treatment significantly increased the contents of catechin, epicatechin, procyanidin B2 and procyanidin B3 at 10, 30 and 50 DAT; in contrast, application of the ethylene receptor inhibitor 1-MCP led to the opposite results. Therefore, ethylene plays a key role in regulating PA synthesis (Fig. 3C-F). Melatonin also significantly increased the content of the four compounds, whereas the application of 1-MCP significantly inhibited the melatonin-induced increases in the content of the four compounds (Fig. 3c-f). Therefore, melatonin regulates accumulation of PAs partially through ethylene production.

### VvMYB14 and VvERF104 activate transcription of *VvMYBPA1* and *VvMYBPA2*, respectively, two key transcription factors involved in regulating PA synthesis

VvMYBPA1 and VvMYBPA2 have been identified as regulators of PA synthesis [6, 7]. Hence, the 1000 bp fragment upstream of the start codon of *VvMYBPA1* and *VvMYBPA2* was used to screen transcription factors via a yeast one-hybrid system (Y1H), and VvMYB14 and VvERF104 were identified as possible regulatory factors of *VvMYBPA1* and *VvMYBPA2*, respectively.

**Figure 4:**
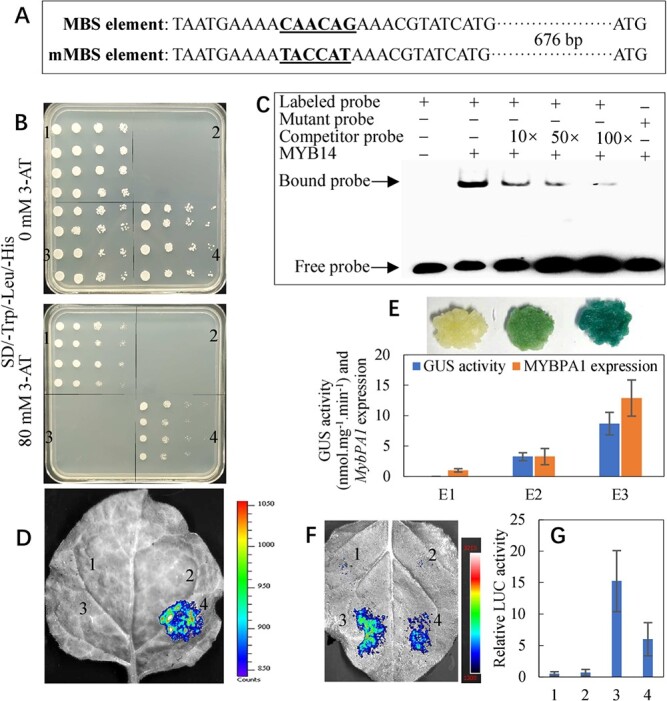
Characterization of the transcriptional activation of *VvMYBPA1* by VvMYB14. (A) Sequence fragments containing MBS or mMBS elements for Y1H and EMSA. (B) Y1H assay. 1) p53::pHis2 + pGADT7-Rec2-p53 (positive controls) [20]; 2) pHis2 + pGADT7; 3) MBS::pHIS2 + pGADT7; 4) MBS::pHIS2 + pGADT7-VvMYB14. In panels 1–4, the yeast cells were diluted 1-, 5-, 50-, and 500-fold, respectively, from left to right. 3-amino-1,2,4-triazole (3-AT) was used as a screening marker. (C) Interaction of the VvMYB14 fusion protein with the DNA probes for MBS or mMBS elements within the *VvMYBPA1* promoter in an EMSA. (D) Representative images of tobacco leaves at 60 h after infiltration. 1) MBS::pGreen П 0800-LUC; 2) MBS::pGreen П 0800-LUC + 35S::pGreen П 62-SK; 3) mMBS::pGreen П 0800-LUC + 35S::pGreen П 62-SK-MYB14; 4) MBS::pGreen П 0800-LUC + 35S::pGreen П 62-SK-MYB14. (E) Histochemical staining and GUS activity determination. (E1) Control; (E2) P_MYBPA1_::MYBPA1-GUS; (E3) P_MYBPA1_::MYPA1-GUS + 35S::MYB14. (F) Representative images of *Nicotiana benthamiana* leaves at 48 h after infiltration are shown. 1) MBS::pGreen П 0800-LUC; 2) MBS::pGreen П 0800-LUC + 35S::pGreen П 62-SK; 3) MBS::pGreen П 0800-LUC + 35S::pGreen П 62-SK-MYB14; and 4) MBS::pGreen П 0800-LUC + 35S::pGreen П 62-SK-MYB14 + 35S::pGreen П 62-SK-TT8 + 35S::pGreen П 62-SK-TTG1. Firefly luciferase activities were quantified and normalized to *Renilla* luciferase activities. Columns 1–4 are defined in Panel F.

Two possible MYB binding sites (MBS and MRE) are predicted in the *VvMYBPA1* promoter (Supplementary Data Fig. S3). In contrast, MBS and MRE were not found in the promoter of *VvMYBPA2* (Supplementary Data Fig. S3). Y1H and electrophoretic mobility shift assays (EMSAs) indicated binding of the VvMYB14 protein to the sequence containing the MBS element within the *VvMYBPA1* promoter (Fig. 4A, B; Fig. 4A, C). In contrast, VvMYB14 did not bind to the fragment containing a mutated MBS element (Fig. 4A, C). Therefore, the VvMYB14 protein specifically binds to the MBS element of the *VvMYBPA1* promoter. On the other hand, Y1H and EMSA results showed very weak binding between VvMYB14 and MRE (Supplementary Data Fig. S4). Collectively, MBS in *VvMYBPA1* is the primary binding site for VvMYB14.

Additionally, *Agrobacterium*-mediated transient expression of the LUC and GUS reporter genes in tobacco leaves and grape calli was performed. Leaves cotransformed with MBS-35 s mini-LUC and 35S-MYB14 showed markedly increased luminescence intensity compared with those transformed with mMBS-35 s mini-LUC and 35S-MYB14 (Fig. 4D). Additionally, grape callus cotransformed with P_MYBPA1_::MYBPA1-GUS (VvMYBPA1-GUS fusion gene driven by the VvMYBPA1 promoter) and 35S::MYB14 showed a darker blue color and higher GUS activity and VvMYBPA1 expression than callus transformed with P_MYBPA1_::MYBPA1-GUS alone (Fig. 4E). Therefore, the VvMYB14 protein acts upstream of *VvMYBPA1* to activate its expression.

Previous studies have shown that the bHLH protein TT8 and WD40 protein TTG1 interact with various R2R3-MYBs and form ternary protein complexes named MBW [19]. However, induction of *MYBPA1* promoter activity by VvMYB14 was not dependent on the presence of TT8 and TTG1 (Fig. 4F, G).

**Figure 5:**
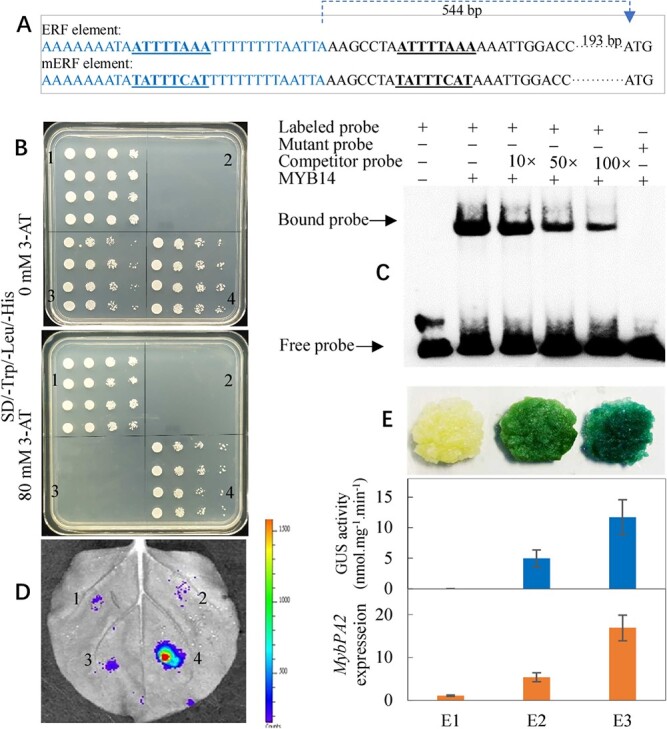
Characterization of the transcriptional activation of *VvMYBPA2* by VvERF104. (A) Two sequence fragments containing ERF or mERF elements, highlighted with different colors, in the promoter of the *VvMYBPA2* promoter were tandemly integrated for Y1H and EMSAs. (B) Y1H assay. 1) p53::pHis2 + pGADT7-Rec2-p53 (positive controls); 2) pHis2 + pGADT7; 3) ERRF::pHIS2 + pGADT7; 4) ERF::pHIS2 + pGADT7-VvERF104. In panels 1–4, the yeast cells were diluted 1-, 5-, 50-, and 500-fold, respectively, from left to right. 3-AT was used as a screening marker. (C) Interaction of the VvERF104 fusion protein with the DNA probes for ERF elements or mERF elements in the VvMYBPA2 promoter in an EMSA. (D) Representative images of tobacco leaves at 60 h after infiltration. 1) ERF::pGreen П 0800-LUC; 2) ERF::pGreen П 0800-LUC + 35S::pGreen П 62-SK; 3) mERF::pGreen П 0800-LUC + 35S::pGreen П 62-SK-ERF104; 4) ERF::pGreen П 0800-LUC + 35S::pGreen П 62-SK-ERF104. (E) Histochemical staining and GUS activity determination. (E1) Control; (E2) P_MYBPA2_::MYPA2-GUS; (E3) P_MYBPA2_::MYPA2-GUS + 35S::ERF104.

Nevertheless, VvERF104 was demonstrated to bind to the ERF element in the *VvMYBPA2* promoter and activate its expression using Y1H, EMSA, and transient expression assays in tobacco leaves and grape calli (Fig. 5A-E).

### VvMYB14 regulates PA accumulation by modifying expression of *VvMYBPA1*, *VvMYBPA2* and *AtTT2* in grape calli and *Arabidopsis* seeds

**Figure 6:**
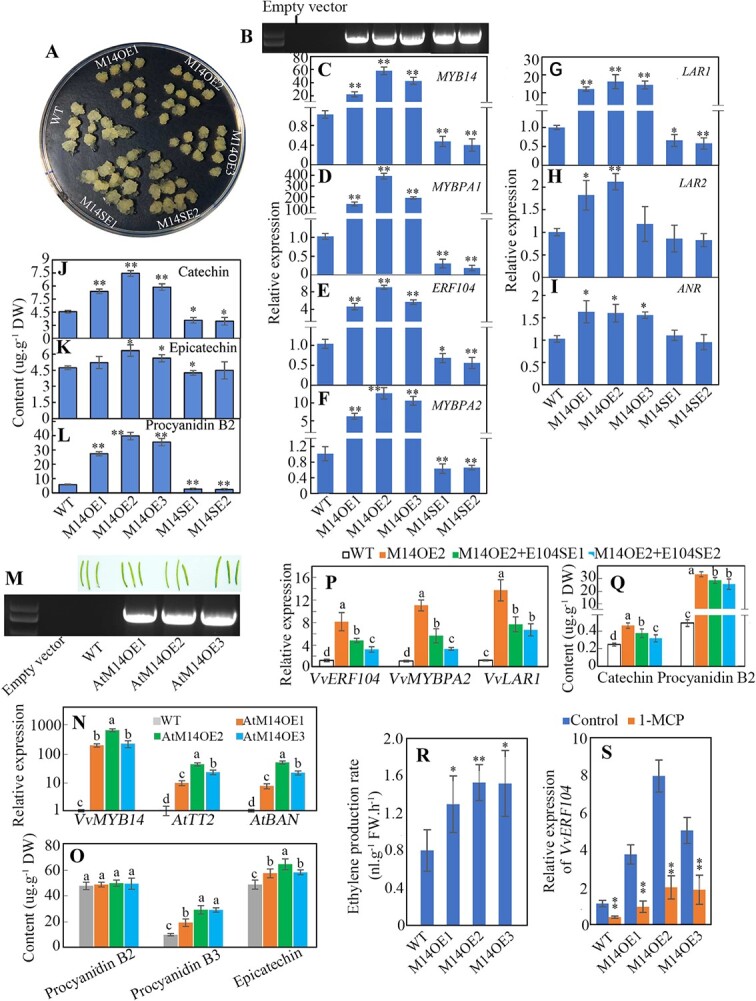
Functional identification of *VvMYB14* in regulating PA synthesis by modifying gene expression. (A) *MYB14*-overexpressing (M14OE) and *MYB14*-suppressing (M14SE) grape calli derived from grape seeds. The photo was taken at 35 days after subculture. (B) PCR identification of transgenic calli using the specific primer pair for the 35S promoter and the *VvMYB14* gene. (C-I) Expression levels of *VvMYB14*, *VvERF104* and other genes related to PA synthesis in WT, M14OE and M14SE grape calli. (J-L) PA content in WT, M14OE and M14SE grape calli. (M) PCR identification of the *VvMYB14*-overexpressing *Arabidopsis* plants using the specific primer pair for the 35S promoter and the *VvMYB14* gene. (N, O) Levels of gene expression (N) and PA accumulation (O) in seeds from the unripe siliques, which are shown in panel M. (P, Q) The expression levels of *VvERF104*, *VvMYBPA2* and *VvLAR1* (P) and content of catechin and procyanidin B2 (Q) in WT, M14OE2 and M14OE2 + E104SE calli. M14OE2 + E104SE calli were obtained by simultaneously overexpressing *VvMYB14* and suppressing *VvERF104* based on M14OE2 calli. (R) Ethylene production level in WT and *VvMYB14*-overexpressing lines. (S) Expression of *VvERF104* in WT and *MYB14*-overexpressing calli in the presence or absence of 1-MCP. The values represent the means ± SD of three replicates. ^*^, Significant difference, P < 0.05; ^**^, highly significant difference, P < 0.01. ns, not significant at P < 0.05. The values indicated by the different lowercase letters are significant at P < 0.05.

To identify the function of *VvMYB14*, transgenic grape seed-derived calli with different levels of *VvMYB14* expression were obtained, including three overexpression lines (M14OE1–3) and two suppression lines (M14SE1–2) (Fig. 6A-C). Overexpression of *VvMYB14* significantly upregulated expression of *VvERF104*, *VvMYBPA2* and particularly *VvMYBPA1*, whereas *VvMYB14* suppression decreased expression of the above three genes (Fig. 6D-F). Additionally, expression of *VvLAR1*, the possible target gene of VvMYBPA1 and VvMYBPA2 [6, 7], was largely increased by *VvMYB14* overexpression. In contrast, two other possible target genes, *VvLAR2* and *VvANR* [6, 7], showed a small increase in gene expression compared to WT (Fig. 6G-I). Except for procyanidin B3, the remaining PAs were detected in grape calli. Contents of catechin and procyanidin B2 were largely increased in *VvMYB14*-overexpressing calli but significantly reduced in *VvMYB14*-suppressed lines*.* In contrast, epicatechin was significantly changed by *VvMYB14* only in M14OE2, M14OE3 and M14SE1 (Fig. 6J-L).

Additionally, three lines of *VvMYB14*-overexpressing *Arabidopsis* plants (AtM14OE1, AtM14OE2 and AtM14OE3) were obtained (Fig. 6M, N) to evaluate their function of this gene in regulating PA biosynthesis in seeds. Expression levels of *AtTT2* (the homologous gene of *VvMYBPA1* and *VvMYBPA2*) [21] and *AtBAN* (the homologous gene of *VvANR*) [22] were largely upregulated, and the contents of procyanidin B3 and epicatechin were significantly increased in seeds from immature siliques of the three lines (Fig. 6N, O).

Moreover, M14OE2 calli were used to obtain grape calli with simultaneous *VvMYB14* overexpression and *VvERF104* suppression (M14OE2 + E104SE1 and M14OE2 + E104SE2). The *VvMYB14* overexpression-induced increases in expression of *VvMYBPA2* and *VvLAR1* and the contents of catechin and procyanidin B2 were significantly reduced by suppression of *VvERF104* (Fig. 6 P, Q). Therefore, VvMYB14 promotes *VvMYBPA2* and *VvLAR1* expression and thereby PA accumulation via VvERF104. Additionally, absence of MBS and MRE excludes the possibility of the interaction between VvMYB14 and the *VvERF104* promoter (Supplementary Data Fig. S5), and*VvMYB14* overexpression increased ethylene production in grape calli (Fig. 6R). These results suggest that VvMYB14 increases *VvERF104* expression by promoting ethylene production. This inference was verified by determination of *VvERF104* expression in *VvMYB14*-overexpressing calli treated with 1-MCP (Fig. 6S).

Taken together, these results suggest that VvMYB14 promotes PA synthesis by directly transactivating *VvMYBPA1* expression or indirectly increasing *VvMYBPA2* expression through enhanced ethylene production. In addition, it was observed that alterations in *VvMYB14* expression affected callus growth (Fig. 6A, Supplementary Data Fig. S6), suggesting the role of *VvMYB14* in regulating growth.

### VvMYB14 mediates melatonin-induced PA biosynthesis

**Figure 7:**
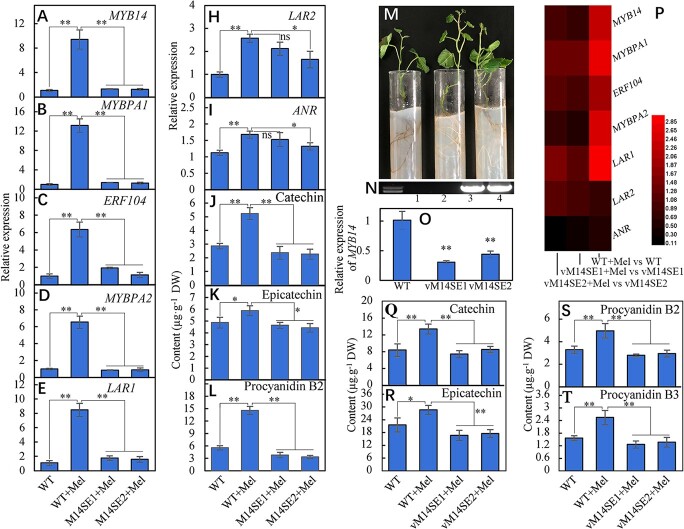
Changes in expression of genes involved in PA synthesis and the PA content in *VvMYB14*-suppressing grape calli and leaves in the presence or absence of melatonin. The data in Panels A to L were from WT and *VvMYB14*-suppressing grape calli (M14SE). The phenotypes of the WT and *VvMYB14*-suppressing grapevines (vM14SE1) are shown in Panel M. PCR identification of *VvMYB14*-suppressing grapevines using the specific primer pair for the 35S promoter and the *VvMYB14* gene is shown in Panel N, in which 1, 2, 3 and 4 represent the empty vector, WT, vM14SE1 and vM14SE1, respectively. The data in Panels O–T are for WT and *VvMYB14*-suppressing grapevines. Mel represents melatonin. ^*^, Significant difference, P < 0.05; ^**^, highly significant difference, P < 0.01. ns, not significant at P < 0.05.

To identify the role of *VvMYB14* in mediating melatonin signaling, *VvMYB14*-suppressed grape calli were treated with melatonin. In WT grape calli, melatonin treatment largely induced expression of *VvMYB14*, *VvMYBPA1*, *VvERF104*, *VvMYBPA2* and *VvLAR1*, whereas melatonin increased expression of *VvLAR2* and *VvANR* to a small extent. These melatonin-induced increases in expression of *VvMYB14*, *VvMYBPA1*, *VvERF104*, *VvMYBPA2* and *VvLAR1* were largely reduced in *VvMYB14*-suppressed calli (Fig. 7A-H). Similarly, the contents of catechin, epicatechin and procyanidin B2 were significantly increased in response to melatonin; in contrast, *VvMYB14* suppression reduced their increase under melatonin treatment (Fig. 7J-L). Additionally, two *VvMYB14*-suppressed grapevines (vM14SE1 and vM14SE2) were obtained to further evaluate the role of *VvMYB14* in mediating melatonin-induced PA accumulation (Fig. 7M-O). In grape leaves, *VvMYB14* suppression significantly reduced the melatonin-induced increases in expression of *VvMYB14*, *VvMYBPA1*, *VvERF104*, *VvMYBPA2* and *VvLAR1* and in the contents of catechin, epicatechin, procyanidin B2 and procyanidin B3 (Fig. 7P–T).

Collectively, melatonin promotes expression of *VvMYBPA1*, *VvERF104*, *VvMYBPA2* and *VvLAR1* via *VvMYB14* and therefore increases PA accumulation. In addition, *VvMYB14*-suppressed vines grew quickly and exhibited more white roots than WT (Fig. 7M; Supplementary Data Fig. S7), suggesting a role for *VvMYB14* in regulating growth.

## Discussion

### Possible roles of melatonin and *VvMYB14* in the PA biosynthesis pathway

The role of melatonin in increasing PA accumulation was indicated by exogenous treatment in seed coats (Fig. 2F-J). In contrast, a high PA content was detected in seeds at 60 DAB, a time when melatonin was undetectable (Fig. 1C, E-H). Although there was a continuous increase in melatonin content and *VvMYB14* expression (Fig. 1B, C), a relatively low content of PAs was detected in seeds at 100 DAB (Fig. 1D-H), suggesting the existence of other negative regulators of PA biosynthesis. Therefore, it is suggested that melatonin acts as a modulator of PA biosynthesis rather than a trigger. Additionally, the contrasting effects of exogenous melatonin treatment on PA accumulation and expression of genes related to PA biosynthesis in seeds and skin (Fig. 2F-J, Supplementary Data Figs. S1 and S2) indicate that the effects of melatonin are complex and may largely depend on the plant tissue. This inference is also supported by other studies showing that melatonin treatment had opposite effects on postharvest ripening of tomato and banana [10, 12].

Overexpression of *VvMYB14* increased accumulation of PA compounds in seed-derived grape calli (Fig. 6J-L) and *Arabidopsis* seeds (Fig. 6O). Similar results were found for *MtMYB14*-overexpressing hairy roots in *M. truncatula* [18]. A phylogenetic tree shows that VvMYB14, MtMYB14 and AtMYB14 belong to the same subgroup (Supplementary Data Fig. S8). Therefore, MYB14 might regulate PA biosynthesis in different species. Notably, *VvMYB14* overexpression led to different effects on PA compound accumulation in grape calli and *Arabidopsis* seeds (Fig. 6J-L, O), which is probably related to the absence of the LAR gene in *Arabidopsis* genome, at least partially [21]. Therefore, the role of MYB14 in regulating PA biosynthesis is affected by its downstream genes. Additionally, *VvMYB14* was strongly induced by melatonin in seed coats (Fig. 2E) and skin [15]. Suppression of *VvMYB14* reduced the effects of melatonin on PA accumulation in grape calli (Fig. 7J-L) and leaves (Fig. 7 P-S). Thus, *VvMYB14* mediates melatonin-induced PA biosynthesis. Notably, *VvMYB14* overexpression largely increased the catechin content in seed-derived grape calli (Fig. 6J); however, the opposite results were found in skin-derived calli [15]. These contrasting results suggest that additional seed-derived factors may be needed to increase PA biosynthesis in grape calli under *VvMYB14* overexpression. The cell-specific function of *VvMYB14* might be associated with the tissue-specific regulation of melatonin, as mentioned above.

### *VvMYBPA1* and *VvLAR1* might be primary regulatory and structural genes, respectively, in the *VvMYB14*-mediated PA biosynthesis pathway

VvMYBPA1 and VvMYBPA2 are two key transcription factors responsible for regulating PA biosynthesis through modification of *VvLAR* and *VvANR* expression [6, 7]. A phylogenetic tree showed that VvMYBPA1 and AtMYB123 (TT2) cluster into the same subgroup (Supplementary Data Fig. S8). In particular, *VvMYBPA1* complements the PA-deficient seed phenotype of the *Arabidopsis tt2* (*Atmyb123*) mutant*,* which shows almost complete loss of PA accumulation in the seed coat [6]. Therefore, VvMYBPA1 is a key transcription factor regulating PA biosynthesis. Additionally, VvMYB14 directly bound to the promoter of *VvMYBPA1* and activated its expression (Fig. 3), and an extreme increase in expression of *VvMYBPA1* and *AtTT2* was detected in *VvMYB14*-overexpressing calli and *Arabidopsis* seeds, respectively (Fig. 6D, N). Accordingly, it is strongly suggested that *VvMYBPA1* might be the key transcription factor in the *VvMYB14*-induced PA biosynthesis pathway. VvMYB14 also increased *VvERF104* expression via ethylene signaling and thereby upregulated *VvMYBPA2* expression (Fig. 6R, S). It has been reported that overexpression of *VvMYBPA2* results in accumulation of *VvMYBPA1* transcripts in grape hairy roots [7]. Collectively, VvMYB14 increases *VvMYBPA1* expression via direct transactivation and indirectly via VvMYBPA2. Notably, VvMYB14 transactivated *VvMYBPA1* in the absence of bHLH and WD40 (Fig. 4F, G). Similarly, it has been reported that VvMYB14, VvMYB15, and VvMYBF1 induce the promoter activity of target genes in a bHLH cofactor-independent manner [16]. Moreover, the protein sequence of VvMYB14 lacks the [D/E]Lx2[R/K]x3Lx6Lx3R motif (Supplementary Data Fig. S9), which is necessary for interaction with bHLHs [16, 23]. These MYBs that lack the bHLH interaction amino acid motif may not require bHLH TFs for their activity.

LAR is predominantly responsible for producing (+)-catechin, and ANR is predominantly responsible for producing (−)-epicatechin [5, 24, 25]. *VvMYB14* overexpression led to much higher expression of *VvLAR1* than that of *VvLAR2* and *VvANR* (Fig. 6G-I), which corresponded to the larger increase in catechin than epicatechin (Fig. 6J, K). Additionally, overexpression of *VvMYBPA1* or *VvMYBPA2* did not result in significant induction of *VvLAR2* [7]. Hence, it is suggested that *LAR1* is the primary structural gene for PA biosynthesis in the *VvMYB14*-mediated pathway. It has also been reported that MtMYB14 strongly induces PA accumulation by activating the promoters of *ANR* and *LAR* in *M. truncatula* hairy roots [16]. Therefore, MYB14 might regulate PA biosynthesis by upregulating *MYBPA1* and *MYBPA2,* thereby increasing expression of *ANR* and *LAR*, or by directly activating *ANR* and *LAR*.

### VvMYB14 serves as a bridge between melatonin and ethylene, affecting PA biosynthesis via ethylene.

Ethylene is an important signaling molecule that plays a key role in regulating fruit maturation and senescence. Ethylene is the dominant regulator of anthocyanin and some other phenolic compound biosynthesis in grape berry skin [15, 26]. However, the specific role of ethylene in modifying PA accumulation is poorly understood. Here, we elucidate the positive influence of ethylene on PA biosynthesis via ethephon and 1-MCP treatment in seed coats (Fig. 3). Melatonin treatment significantly increases ethylene production levels in different tissues of grapes, including Merlot grape seeds (Fig. 3A, B) and skin [15], whole Moldova berries [11], and the roots and leaves of Crimson seedless grapes [27]. Similar results have been found in tomato fruits [10]. Therefore, it is inferred that melatonin promotes PA biosynthesis through ethylene, at least partially. This inference was verified by treating seed coats with melatonin plus 1-MCP (Fig. 3C-F). However, the ethylene production rate and melatonin content showed different patterns in seeds during berry ripening (Fig. 2D and Fig. 3B), suggesting that melatonin is not the sole signaling molecule that regulates ethylene production. For example, reciprocity between ABA and ethylene exists in grape fruits [28].

*VvMYB14* is strongly induced by melatonin (Fig. 2D), and a 580-bp core region responding to melatonin has been identified [15]. VvMYB14 promotes ethylene production by transactivating *VvACS1* expression by binding to its promoter [27]. Therefore, VvMYB14 mediates melatonin-induced ethylene production. In ethylene signaling, ERFs are downstream transcription factors that activate expression of ethylene-responsive genes and are involved in the regulation of numerous developmental processes and stress tolerance. In particular, MdERF1B is reported to regulate anthocyanin and PA biosynthesis in apple [29]. In the present study, VvERF104 was found to function downstream of VvMYB14 to increase PA biosynthesis by transactivating expression of *VvMYBPA2* (Fig. 5). ERF104 may be involved in pathogen responses [30]. In fact, PAs, as astringent compounds, are considered to be involved in defense against herbivores and pathogens in leaves and unripe fruits [31]. These results suggest that ERF104 might regulate stress tolerance by increasing PA biosynthesis.

Collectively, melatonin increases ethylene production by inducing *VvMYB14* expression, and the increase in ethylene promotes PA biosynthesis through VvERF104-activated *VvMYPA2* expression.

### MYB14 might possess multiple functions, including growth regulation

In this study, we also found that *VvMYB14* overexpression inhibited callus growth and that its suppression promoted the growth of calli and vines and caused vines to produce more white roots (Fig. 6A; Fig. 7M; Supplementary Data Figs. S6 and S7). It has also been reported that *MYB14* is related to biosynthesis of stilbene in grapevine [16] and starch in the maize endosperm [32]. Some direct target genes or downstream genes regulated by MYB14 have been identified, including *STS*s in grape [16] and six starch-synthesizing genes and 12 genes involved in the general phenylpropanoid pathway in lotus [33]. Therefore, *MYB14* might play a broad role in regulating metabolism and growth.

Regarding the growth inhibition caused by *VvMYB14* overexpression, we inferred that it might be related to high-level PAs that excessively accumulated and produced toxic effects in *VvMYB14*-overexpressing cells. The toxic effect of PAs is also shown by results that large accumulation of PAs in the roots and leaves of transgenic grapevine or *Arabidopsis* leads to growth abnormalities and even death of the plants [6, 34]. Alternatively, *VvMYB14* overexpression increases the level of ethylene, which is a pleiotropic molecule with diverse functions in plants, including growth inhibition [35]. Additionally, *ERF104* overexpression largely increases expression of four auxin-responsive proteins and one auxin efflux carrier in *Arabidopsis* [30], suggesting that *MYB14* affects auxin signaling and therefore regulates growth.

**Figure 8:**
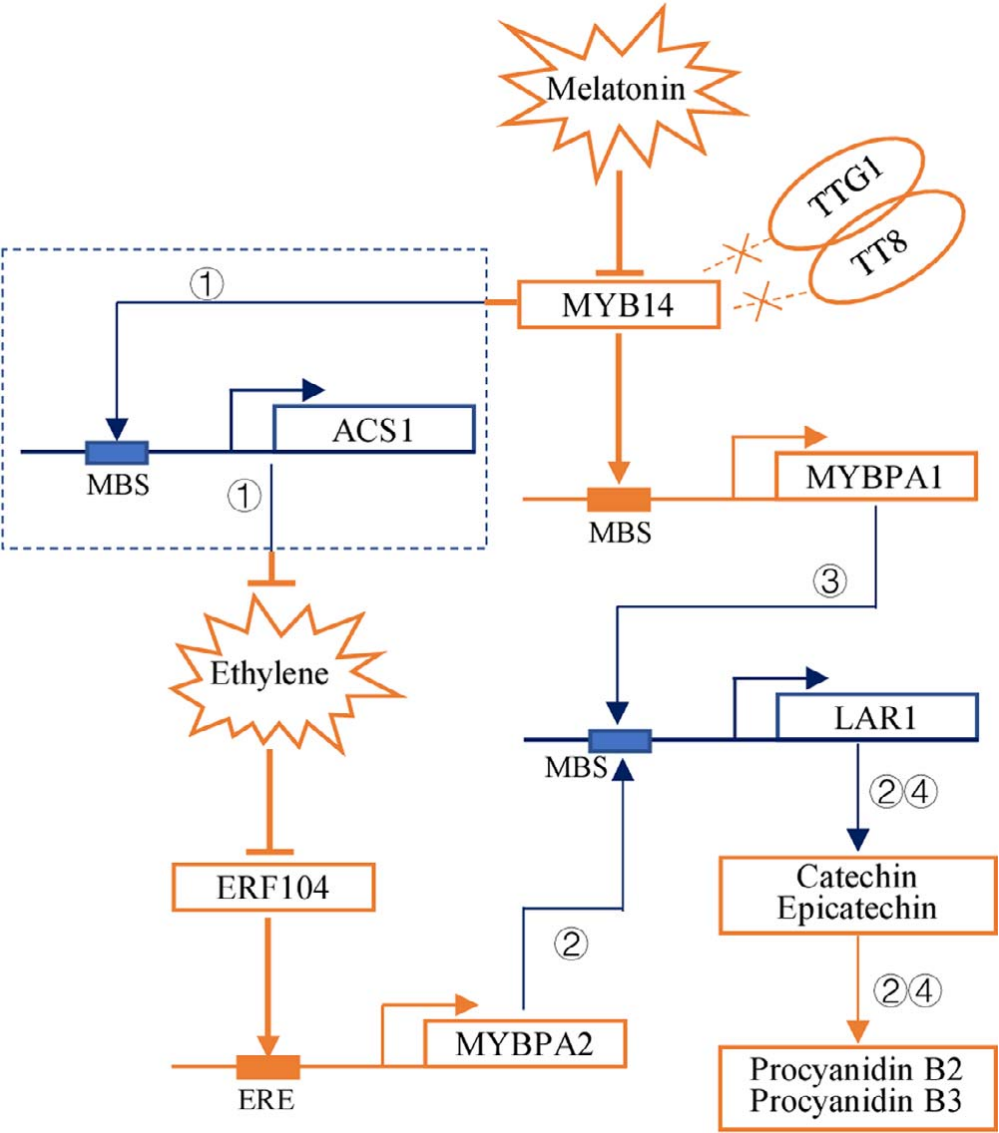
Model of the regulation of PAs by melatonin via the *VvMYB14*-mediated pathway. The red lines and arrows indicate the pathway verified in grapevine in the present work; conversion of catechin/epicatechin into procyanidin B2/procyanidin B3 refers to previous studies [6, 18]. The blue lines and arrows indicate pathways that have been previously reported in grapevines: ① Xu *et al*., 2019 [24]; ② Bogs *et al*., 2007 [6]; ③ Terrier *et al*., 2009 [7]; and ④ Liu *et al*., 2014 [18]. Cross marks indicate that VvMYB14 functions independently of VvTTG1 and VvTT8.

Taken together, these results indicate that *VvMYB14* expression and melatonin accumulation continuously increase in seeds during berry ripening. VvMYB14 binds to the promoter of *VvMYBPA1* and activates its expression. VvMYB14 also increases *VvERF104* expression by promoting ethylene production and thereby enhances *VvMYBPA2* expression. *VvMYB14* overexpression increases PA accumulation in grape seed-derived calli and *Arabidopsis* seeds. Exogenous melatonin treatments largely increase *VvMYB14* expression and PA accumulation in seed coats. Melatonin-induced increases in PA compounds were significantly reduced by *VvMYB14* suppression in grape calli and leaves. Therefore, it is most likely that melatonin promotes PA biosynthesis by upregulating expression of *VvMYB14* and thereby *VvMYBPA1* and *VvMYBPA2* in grape seeds (Fig. 8).

## Materials and methods

### Plant materials and growth conditions

Skin and seeds from Merlot grape (*Vitis vinifera*) berries at different developmental stages were collected to determine gene expression and melatonin and PA contents. Preveraison Merlot grape berries at 60 days after bloom (DAB) were treated with 50 μM melatonin, which was verified to effectively increase the melatonin level in berries and affect berry ripening and secondary metabolite accumulation [15, 17], 200 mg. l^−1^ ethephon, 10 μl. l^−1^ 1-MCP, or 50 μM melatonin plus 10 μl. l^−1^ 1-MCP. Clusters were soaked in the above solutions with 0.05% Triton X-100 and in 0.05% Triton X-100 alone (control). Each treatment consisted of three replications, and each replication consisted of 3 vines (approximately 24 clusters). Berries were randomly sampled from each cluster for subsequent experiments.

Seeds were cross-cut into halves and cultured on MS media containing 1.2 mg. l^−1^ TDZ and 0.12 mg. l^−1^ IBA to induce nonembryogenic calli. The resulting calli were subcultured on MS medium containing 30 g. l^−1^ sucrose, 7 g. l^−1^ agar, 0.60 g. l^−1^ 2-(N-morpholino) ethanesulfonic acid, 2.5 mg. l^−1^ thidiazuron (TDZ), and 8 mg. l^−1^ picloram at 28°C. Embryogenic calli of Thompson Seedless anthers were used for gene transformation. Embryogenic calli were subcultured on MS medium containing 3 g. L^−1^ Phytagel, 45 g. L^−1^ sucrose, and 1.2 g. L^−1^ activated charcoal.

### Determination of total anthocyanins, total soluble solids (TSS), and titratable acid contents

Total anthocyanins of berry skin were extracted and determined using the spectrophotometric pH differential method as described in a previous study [17]. Fresh pulp was ground and filtered, and the filtrate was used to determine TSS and titratable acid. The TSS content was determined using a digital display sugar meter, and titratable acid was measured by titration of the filtrate with 0.1 M NaOH to pH 8.3 [15].

### Melatonin extraction and determination using UPLC–MS

Extraction and determination of melatonin were performed according to our previous study [17] with minor modifications. Briefly, two grams of ground tissue was preliminarily extracted two times using methanol by sonicating for 20 min each time. The combined supernatants were centrifuged at 8000 rpm for 10 min and then evaporated to dryness at 30°C. The residue was dissolved in methanol and then purified using a ProElut™ C_18_ solid-phase extraction (SPE) cartridge (Dikma, China). The purified samples were separated and detected by UHPLC–MS (Waters, Milford, MA, USA) equipped with a BEH C_18_ column and a QTof-micro mass spectrometer. The same UHPLC and MS conditions as in our previous study were applied in this study. Melatonin was quantified using the external calibration curve of a melatonin standard.

### PA histochemistry, extraction and determination

For detection of PAs, seed cross sections were stained with DMACA as described by Feucht and Polster [36]. PA was extracted according to our previous study [37], and the process was similar to the melatonin extraction but with the following changes: methanol was replaced by acidified methanol (0.1% HCl, v/v). Samples without SPE purification were directly used for total PA determination. Purified samples were used for determination of PA compounds. The total PA content was measured using a vanillin assay [38]. Briefly, 200 μl of extract was added to a reaction solution with 500 μl of 1% (w/v) vanillin in methanol and 500 μl of 25% (v/v) H_2_SO_4_ in methanol; after 15 minutes at 30°C, the A500 value was measured by a spectrophotometer. PA compounds were detected using a Dionex Ultimate 3000 UHPLC system and a ESI-triple quadrupole mass spectrometer (Thermo Fisher Scientific, San Jose, CA, USA). The UHPLC system was equipped with a reversed-phase C_18_ analytical column (100 mm × 2.1 mm, 1.9 μm). The detailed parameters and conditions of HPLC and MS were fully described in our previous study [37]. The amount of PA compounds was quantified using the external calibration curves of corresponding standards.

### Determination of ACC content and ethylene production rate

Extraction and determination of ACC were performed based on a previously described method [39]. Ethylene production rate was determined using a Shimadzu GC-9A gas chromatograph (Kyoto, Japan) equipped with a flame ionization detector and a GDX-502 column as described in a previous study [17].

### Yeast one-hybrid (Y1H) assays and electrophoretic mobility shift assays (EMSAs)

For Y1H assays, sequences including the MBS or ERE elements from the promoter of *VvMYBPA1* or *VvMYBPA2* were cloned into the pHis2 vector. Mutant MBS or ERE elements were used as a negative control. The ORF of *VvMYB14* or *VvERF104* was cloned into the pGADT7 vector. The resulting plasmid was integrated into yeast strain Y1HGold. Y1H assay was conducted using a Matchmaker™ Gold Yeast One-Hybrid Library Screening kit from Clontech (Mountain View, CA, USA).

For EMSA, the recombinant VvMYB14- or VvERF104-His protein was expressed using a pET-32a vector and the soluble proteins were purified via a BeaverBeads IDA-Nickel kit (Beaver, BioBay, China). DNA probes containing an MBS or ERE element were synthesized and labeled with biotin. EMSAs were conducted according to the user manual of the LightShift Chemiluminescent EMSA kit (Thermo Fisher Scientific, Waltham, MA, USA). The primers used in Y1H assays and EMSA are listed in Supplementary Data Table S1.

### Transient cotransformation in tobacco leaves and grape calli

The ORFs of *VvMYB14* and *VvERF104* were inserted into the pGreenII 62-SK vector, and the promoter fragments of *VvMYBPA1* and *VvMYBPA2* containing MBS and ERE elements, respectively, were cloned into the pGreenII 0800-LUC vector. The plasmids were transiently transformed into tobacco leaves using a previously described method [40].

The *VvMYB14* ORF was inserted into the pRI101-AN vector to produce the construct *35S::MYB14*. The 35S promoter within pRI101-GUS was replaced with the promoter of *VvMYBPA1* or *VvMYBPA2*, 1000 bp upstream of the start codon, and the *VvMYBPA1* or *VvMYBPA2* ORF was inserted upstream of GUS, yielding the construct *P_MYBPA1/MYBPA2_::MYBPA1/MYBPA2-GUS*. The plasmids were transformed into nonembryogenic grape calli according to the method of Xu *et al*. [27] Histochemical staining and activity determination of GUS were conducted according to a previously reported method [41].

### Transformation of *VvMYB14* or *VvERF104* into merlot seed-derived calli and grapevines and *Arabidopsis* plants

The construct *35S::MYB14* involving the pRI101-AN vector mentioned above was used for sense overexpression. The 3’-UTR sequence of *VvMYB14* was inserted into the pRI101-AN vector, and the obtained construct was used for antisense suppression. Additionally, the 3’-UTR sequence of *VvERF104* was inserted into the pHB vector with hygromycin resistance for cotransformation with *VvMYB14*. Transgenic grape calli were obtained using an *Agrobacterium*-mediated transformation method [42].

Additionally, the pRI101-AN vector containing the 3’-UTR sequence of *VvMYB14* was transferred into grapevine embryogenic calli using the *Agrobacterium* (GV3101)-mediated method [20]. Briefly, the calli were soaked in bacterial solution for 20 min with gentle shaking. Cocultivation in solid MS medium containing 15 mg. l^−1^ acetosyringone and 2% sucrose was performed for 3 days at 28°C in the dark. The calli were screened on KBN delayed-screening medium containing 250 mg. l^−1^ cefotaxime for 4 weeks and then on X3 delayed-screening medium containing 250 mg. l^−1^ cefotaxime for 1 week. The transformed calli were cultured in resistance screening medium containing 250 mg. l^−1^ cefotaxime and 75 mg. l^−1^ kanamycin until germinated embryos developed. The embryos were then cultured in germination medium under light until true leaves appeared, and the plantlets were transferred to rooting medium containing 1 mg. l^−1^ IBA.

For *Arabidopsis* transformation, the pRI101-AN vector containing *35S::MYB14* was transferred into Columbia-O by the *Agrobacterium*-mediated floral dip method [43]. All of the above transgenic calli or plants were verified by PCR identification using the specific primer pair for the 35S promoter and the *VvMYB14* gene and determination of the *VvMYB14* expression level.

### Real-time quantitative PCR

Real-time quantitative PCR was performed using SYBR Green Master Mix (SYBR Premix EX TaqTM, Dalian, China) on an ABI7500 qRT–PCR instrument (ABI, MA, USA), and the primers used are listed in Supplementary Data Table S1.

## Acknowledgments

This study was financially supported by National Key R&D Program of China (2018YFD1000200), Agriculture Improved Variety Project of Shandong Province (2020LZGC008), Major Project of Science and Technology of Shandong Province (2022CXGC010605), Fruit Industrial Technology System of Shandong Province (SDAIT-06-03) and the National Natural Science Foundation of China (31872068 and 32072537).

## Author contributions

Y.Y. and X.Z. conceived and designed the research; X.Z., W.M., F. W. and Z.F. performed the experiments; X.G. and S.G. analyzed the data; and Y.Y. wrote the manuscript. All authors read and approved the manuscript.

## Data availability

All the experimental data are available and accessible via the main text and/or the supplemental data.

## Conflict of interest

The authors declare that they have no conflicts of interest.

## Supplementary data

Supplementary data is available at *Horticulture Research* online.

## Supplementary Material

Web_Material_uhac274
