# Supplementary material for: VvMYB14 participates in melatonin-induced proanthocyanidin biosynthesis by upregulating expression of VvMYBPA1 and VvMYBPA2 in grape seeds
Source: Hortic Res. 2022 Dec 7;10(2):uhac274. doi: 10.1093/hr/uhac274 (PMC10390852; doi:10.1093/hr/uhac274)
Supplement: Web_Material_uhac274 [file web_material_uhac27.docx]

**Supplementary Data**

**Table S1** Primers used in this study.

The gene ID is derived from the grape genome (<http://genomes.cribi.unipd.it/grape/>). The restriction digestion sites are underlined.

| **Gene_symbol and id** | **Forward primer (5'-3')** | **Reverse primer (5'-3')** | **Notes** |
| --- | --- | --- | --- |
| VvMYB14  VIT_207s0005g03340 | TGAAAGGCTGGGCAATAGAT | TGGGGTTTTGTTGTTTTTCG | qRT-PCR |
|  | GAATTCATGGGGAGAGCTCCTTGTTG | GGATCCTCATATTTCTGATAATTCATGC | Amplifying the ORF of *VvMYB14* to construct the pGADT7 vector |
|  | GGATCCATGGGGAGAGCTCCTTGTTG | AAGCTTTCATATTTCTGATAATTCATGC | Amplifying the ORF of *VvMYB14* for EMSA experiment |
|  | GTCGACATGGGGAGAGCTCCTTGTTG | GGATCCTCATATTTCTGATAATTCATGC | Amplifying the ORF of *VvMYB14* to construct the pRI101-AN vector |
|  | GGATCCGCTCCTTGTTGTGAGAAGA | GTCGACACCATCTATTGCCCAGCCTT | Amplifying the 3́-UTR sequence of *VvMYB14* to construct the pRI101-AN vector |
|  | GGATCCATGGGGAGAGCTCCTTGTTG | GGATCCTCATATTTCTGATAATTCATGC | Amplifying the ORF of *VvMYB14* to construct the pGreenII 62-SK vector |
| VvERF104  VIT_216s0013g01080 | TTGAAGCCGCCAGAGGTTAT | CCTTTATCTCCGGTTGTTTGC | qRT-PCR |
|  | GAATTCATGGGAGATGAAGCTTCATCAC | GGATCCTTAAGTTACGAGCCGAGAATATC | Amplifying the ORF of *VvERF104* to construct the pGADT7 vector |
|  | GGATCCATGGGAGATGAAGCTTCATCACTG | AAGCTTTTAAGTTACGAGCCGAGAATATCCA | Amplifying the ORF of *VvERF104* for EMSA experiment |
|  | GGATTCAGGGCGTTGGAAGAAAGCCG | AAGCTTAAATAATGGGACAATTATGAT | Amplifying the 3́-UTR sequence of *VvERF104* to construct the pHB vector |
|  | GGATTCATGGGAGATGAAGCTTCATCACTG | GGATTCTTAAGTTACGAGCCGAGAATATCCA | Amplifying the ORF of *VvERF104* to construct the pGreenII 62-SK vector |
| VvMYBPA1  VIT_215s0046g00170.1 | GGATCCGTTGTTTGACAAAATTTAAGAAAT | GGATCCATTTCTTAAATTTTGTCAAACAAC | Amplifying the promoter of *VvMYBPA1* to replace the 35 S promoter within pRI101-GUS, yielding the construct of P_MYBPA1_-GUS. |
|  | AACTACTGGAACACCCATCTG | ATTTTCTTGGTCCTTGCTGA | qRT-PCR |
| VvMYBPA2  VIT_211s0016g01320.1 | GGATTCGAGAGTCTTCATGAAATTGAC | GGATTCTTATATTCTCTACAAAAAGTCTCTC | Amplifying the promoter of *VvMYBPA2* to replace the 35 S promoter within pRI101-GUS, yielding the construct of P_MYBPA2_-GUS. |
|  | GACATTGGCGACCTCCTTAC | CGAGAAAATCGGAACAAGGA | qRT-PCR |
| VvLAR1 VIT_01s0011g02960 | ATCGGGCATACCCTTCACTTAT | GACATCGTCCACTGTTTTCATC | qRT-PCR |
| VvLAR2 VIT_17s0000g04150 | ATCCCCTACACCTACATCTGCT | ATTTGTTCACTGTTCGATCGTC | qRT-PCR |
| VvANR VIT_02s0025g01260 | TTGTTGCATCTTTGCTGGTTAA | CTAGGAGGTGAGAGACTTTTTT | qRT-PCR |
| VvTT8 VIT_207s0104g00090.1 | GAATTCATGGCTGCGCCGCCGAAT | GAATTCGTACTGGGGTATTATTTGGTTTATTGC | Amplifying the ORF of *VvTT8* to construct the pGADT7 vector |
| VvTTG1  VIT_16s0098g00870 | GAATTCATGGAGAGATCAAGCCAAGAATCC | GAATTCAACTTTAAGAAGCTGCAGTTTGTTGG | Amplifying the ORF of *VvTT1* to construct the pGADT7 vector |
| AtTT2  AT5G35550 | GGCGAAGGCAAATGGAGCACT | CCAAGAAGATTATGGAGACGG | qRT-PCR |
| AtBAN  AT1G61720 | ATGGACCAGACTCTTACACACA | TGAGCTATTTTCTTCTCGTTTT | qRT-PCR |
|  | GGATCCGCTCCTTGTTGTGAGAAGA | CAGGAAACAGCTATGAC(M13R) | Amplifying the fragment of tje 35 S promoter and *VvMYB14* to identify *VvMYB14-*suppressing calli or grapevines |
|  | GACGCACAATCCCACTATCC(35S) | GGATCCTCATATTTCTGATAATTCATGC | Amplifying the fragment of the 35 S promoter and *VvMYB14* to identify *VvMYB14-*overexpressing calli or *Arabidopsis* plants |

**Fig. S1 Changes in expression of genes related to PA biosynthesis in the seed coats of melatonin-treated berries**. The fold change from qRT–PCR was calculated by comparing the relative expression values of the selected genes in the seed coats of melatonin-treated berries and control seed coats.


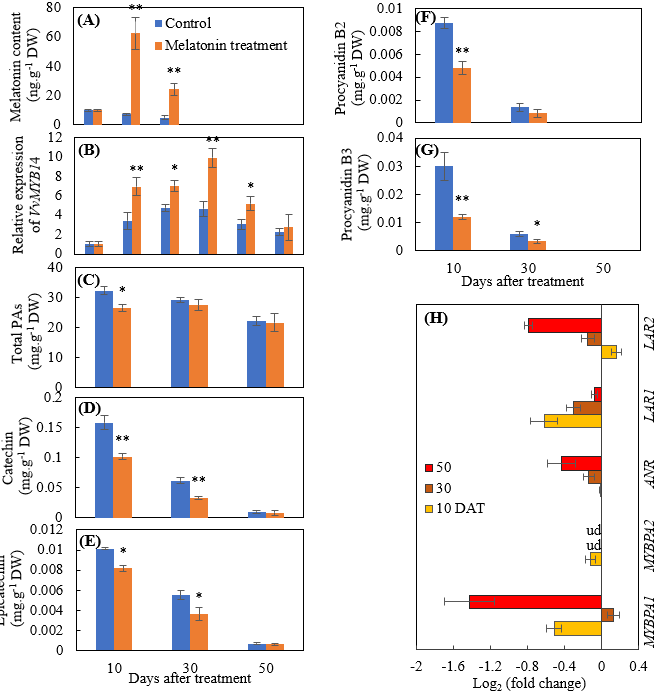


**Fig. S2 Changes in contents of melatonin and PAs and expression of *VvMYB14* and PA biosynthesis-related genes in berry skin under melatonin treatment**. The berries were treated at 60 DAB, corresponding to 0 DAT. Melatonin content (A) and *VvMYB14* expression (B) were detected at 5 time points after melatonin treatment. PA content (C-G) and the expression of the genes related to PA biosynthesis were determined at 10, 30 and 50 DAT. In panel H, fold change from qRT-PCR is calculated by comparing the relative expression values of the selected genes in the melatonin-treated berry skin and control skin. The values represent the mean ± SD of three replicates. *, significant difference, P < 0.05; **, highly significant difference, P < 0.01. ns, not significant at P < 0.05; ud, undetectable.

VvMYBPA1 CCATGGGTAAATAAAT--AAATAGAACGTGCTCCTGTAGTATCATTTTGCATAAATTTTA 58

VvMYBPA2 ---TTGAGAGAGAGAGAGAGAGAGAATGAGCCAAATATAAAAAATATTGCCCCACGCTGA 57

* * * * * * * * **** * ** * ** **** * * *

VvMYBPA1 TGCACGTGAAT--CCCATTAT---------AAAATTAAAAAAATTTATAAATTATG---- 103

VvMYBPA2 TGAAAATGAAAGTGACATAATTTGAGAGAGAGAGAGAGAGAGAGAGAGAGAGTAGGTGAA 117

** * **** *** ** * * * * * * * * * ** *

VvMYBPA1 CTTTAAAAGTATAATTCCTAAATCGTGGGTTTTTCTT-TTTATCAGACTTTTTGTTTTCA 162

VvMYBPA2 ATAGAGAGGTAGATGAAATCATGCGGTGCATTGAAGGATAGTTGAACAAAAATGTGATAG 177

* * * *** * * * ** * ** * * * *** *

VvMYBPA1 TGAATTATAAAATGGTCATAATTATCT--TTGGGACACCTTTATGGGATTCTT------- 213

VvMYBPA2 CAACCTCTAGGGTTCTAAAATTTTTTAATAAAAAGGAACTCTATCTACTTCCAGCGAGGT 237

* * ** * * * * ** * * ** *** ***

VvMYBPA1 --CAACGATACTATAATCTTATGAGATTTCCTTGTACGCAATTATTTCTTTTTTTCTTCT 271

VvMYBPA2 AGCAATATGAAAAAAGACTCTTTAGATCTTCATGTTTGGACCAGTGGTTT-------TCT 290

*** * * * ** * **** * * *** * * * ** ***

VvMYBPA1 TTTAGTTTCCAATAATTATTGATTATATTCTTCCATCATATGCTAAGTTTCAATCTGATA 331

VvMYBPA2 TTTTCTTTCAAATTTTTAGT----GTAGT--ATAAGCATGTGAA-----TCACGTTAG-A 338

*** **** *** *** * ** * * *** ** *** * *

VvMYBPA1 AGATCTTGACACTCCTTGTCAATTTTG----GTGTCGAGACTTTTCTTTATTTCTACTAC 387

VvMYBPA2 AAAATGGGCCCTCCTATGTCTATCATTCATTTATTTGTGATCGTGCATGCATGCTGGTA- 397

* * * * * **** ** * * * ** * * * * ** **

VvMYBPA1 TTCTCAACTCTAATTATTCTTCAATTATGTATCCTTTTTGTCCTTTTAAATCTGCATAAA 447

VvMYBPA2 ---AGAGATATGCTTTAGGTTTTTTTATGACACTGGTAGAACCAAGTGATGGTTTAAAAA 454

* * * ** ** ***** * * ** * * * * ***

VvMYBPA1 CCAACTAAAATTAATGTGCTTAATATATGTGGTTGGTTGGAATTGTAG----TTCAATCC 503

VvMYBPA2 AAATCCAGAAAAGAATATGTTATGAAATTTGTTTTTTAGTAAATATTTTCTCTACAAATA 514

* * * ** * *** * ** ** ** * * ** * * * ***

VvMYBPA1 ACTTTTTTTTTTTATAATATATTTTATGTATTACGATAATATTCCAAGGTTTTTAAGTCA 563

VvMYBPA2 AGTATTTTTGGAAAAATTATAAAGTGTTTATTCAAAATATTATAAATGATTTTTTATTTT 574

* * ***** * * **** * * **** * ** * * * ***** * *

VvMYBPA1 TAA-----------------------------------GATATTAATATGACATATACCT 588

VvMYBPA2 TAAATTTTATCAAATATCTATTTTTTTTTTTTTTTTATGTTGAAAGTGTTTTCTAAAAAT 634

*** * * * * * ** * *

VvMYBPA1 AACGTT------AATCATTACCTATTTGGGTTTATTGACA-----------AATGTTCAA 631

VvMYBPA2 AATGTTAAATAAGCTCTTAAAATACATGTGATTATGCTCTCTTTTTTTAAGTGTGTTTTC 694

** *** ** * * ** ** * **** * ****

VvMYBPA1 TTTTCCTTCACAACGCTAAATCATCACCAAAATGATAAAAGAAAATAAACCATTAATACA 691

VvMYBPA2 TAAAAATATATTAAGAAAAATCATTTATCAGATGCTTCTAAAAAATGA---TTTTAAACT 751

* * * * * ******* * *** * * ***** * ** * **

VvMYBPA1 TATCTTAATCTTCGAGCCTATTTAGCAATGATTTAAA------AAATTATTTTTAGTTAA 745

VvMYBPA2 TTT------TTTCAAGCACTTTTTTTTTTCTTTTTTTCTTTTTTTACACTTTCTATATT- 804

* * *** *** *** * *** * *** ** *

VvMYBPA1 AAATTTGTTTTTGAAAAAGAAAATAAAATTAGTTGTTTGACAAAATTTAAGAAATATTTT 805

VvMYBPA2 AGAAGTATTTTTTAAAAAAACAATATAGTGATTAGAGTGCGTAGATTTAAGTCATGAAA- 863

* * * ***** ***** * **** * * * * * ** * ******* **

VvMYBPA1 TAAAAATCTAAAAAGTCGTTTGTAACGTAGAAAACTTATTTATAAGCTATATTTGGTTTG 865

VvMYBPA2 ------------CATTGAATTA--TGGAGGAGATCTTGTGCAGATG-------------T 896

* * ** * ** * *** * * * *

VvMYBPA1 TGAAAAGCATAAAAAAAAATGGTTTTCTTATATTT----AGTTTCATTGTAGAAAATATT 921

VvMYBPA2 TGAAATGTGTATACAAAAATTTGCGAGTTGAAGCGGAGACAATTGTTGGTGGATGAGAGT 956

***** * ** * ****** ** * ** * ** ** * * *

VvMYBPA1 TTTTTTTAAAATTATTCAATCTTTATATAATAAAAAAAATAAGTAAAATGATTTTGAATA 981

VvMYBPA2 CTTCATGAAATTGACTGATACA-------------------------TGGTGTTTGGCTG 991

** * *** * * * * * * **** *

VvMYBPA1 AACATATAAAAATAATTTATTAAATTCAAATTTAACTTTTGTTTTCTTTTGTTTTACATT 1041

VvMYBPA2 CTGGGAAA------TTGAAAGAAAGTTGAATTTGGATGCTGATTCTCACTGTCAAGCC-- 1043

* * * * *** * ***** * ** ** *** *

VvMYBPA1 TCATTCTTTATTTTCTTTTTTTCGTATTTGTTCTCAAAATTTTCAAAACCAAACATAGTC 1101

VvMYBPA2 --A-----------------------CATG--GTGGGAACTTTAATT-GCCACCA--GTC 1073

* ** * ** *** * * * ** ***

VvMYBPA1 CTAATGTCTTTTCAAGTGATTATTTTAAAAACACTTTTATTAAAAACACTTCAAACAAAT 1161

VvMYBPA2 AGTGAGGTTCTTTAAGTGTAAATACTGCACATGCTTCAAACAAA-GCCCTCCTATGCAAA 1132

* * ** ***** ** * * * *** * *** * ** * * **

VvMYBPA1 ATTCTTGAAATGTACAAGTGTAATTTATAAATTTGCATTAAAAACACTTTAAACAAATAT 1221

VvMYBPA2 ACTTTTGAA-------GATGGGACATATATATGTATATCATAATCTCAGAAGATAGAAAT 1185

* * ***** ** * **** ** * ** * ** * * * * * * **

VvMYBPA1 TTTTGAAACG--------TACAAGTGTAATTTGTACATTTGCAAAAATTCAATGAAGGAA 1273

VvMYBPA2 GATTAAATTAATATATAGAATCTTACTCATATGTAAGGATCTGAACACTTAATAAAATAA 1245

** ** * * ** **** * ** * * *** ** **

VvMYBPA1 ACTTTTAAACGAAAAGGAAATCATCGTCAAAATGAT---AATGA-AAA-------CAACA 1322

VvMYBPA2 AATGATATGAGATTTATGTACCTAAGTCATAATAATGGATTTGGTGGACCGATTCAATTT 1305

* * ** ** * * **** *** ** ** * *

VvMYBPA1 GAAACGTATCATGGAGGGTGATGGTTGATCAATATGGTATCGGATGATCATAGTTTTACT 1382

VvMYBPA2 TGACTGTAAGGTACATGGTGGTAGTTACATTTTGTAGCATCTTATAATCTTGAATTAACA 1365

* *** * * **** * *** * * * *** ** *** * ** **

VvMYBPA1 CTCAAACGTAACCTAAATAATTGTCGGAAACGACAATAGAAACAAAGGGGGGGGGGGGTT 1442

VvMYBPA2 ATTGA----GAAATAATTGAATGTTCGAACAATATTTTTTAAAAA-ATTA------TATT 1414

* * * *** * * *** *** * ** ** **

VvMYBPA1 AGAGCCACAATGATGGGTTGGTAAATATCTCCAGCCTCAAATGAAGGAGAAGAAGAAACA 1502

VvMYBPA2 ATAGTAAAAAAAATAATTTTAAATTTTTTTTAATTAATCAAAGAAATCCCATGGG----- 1469

* ** * ** ** ** * * * * * ** *** * *

VvMYBPA1 GTTTGCTTTTTTCTTTGGGAAAGGACACTCGTATCGACGTTGACTAATGAGCTTTTTCCA 1562

VvMYBPA2 -ATTGAGCTCCATTTTAATCAAATTTGCTTAGGTTTTTGATGGATTTTTTTTTTTTTTCA 1528

*** * *** ** ** * * ** * * ***** **

VvMYBPA1 CATAATCCATGGTCGTTATTGAACTGTCACACGGAGCCAACACTGTGTATGGATGGG--- 1619

VvMYBPA2 CATCTTCAAAAGCTCTTACCAATGAGTAATGGTGACCATTAACGGTACATGGAAGAGTTC 1588

*** ** * * *** * ** * ** * ** ** ***** * *

VvMYBPA1 -----------CAGGAAGAGAAGAAGGG------------TAGATGAAGAGGAGTTGTTG 1656

VvMYBPA2 TACATCGTGGGCTGGTGCATCAAATCACTATACTTACGAGTGATTTCAGAAATATTTGTG 1648

* ** * * * * * *** ** **

VvMYBPA1 AGAT--ATC-----ATGCACAAACCAAACTTTATGCCCAAAAGGAGA-GA---GAAAAGA 1705

VvMYBPA2 TGAATGATGGTGACAGAAATAATTCATAGCTGGTACCTCTGCGGCACTGCCCATTATTTT 1708

** ** * * ** ** * * * ** ** * *

VvMYBPA1 ATAAAAAAAGAAGAAGGGAATTCAATATTTATTATCAATGGTTTCTAGTGTGAAAAAAAG 1765

VvMYBPA2 ATTCAACTTGAAGATACACTTTCACTTAT--TTATTTATTTATAGAAGTATGAGGTCAAA 1766

** ** ***** **** * * **** ** * *** *** **

VvMYBPA1 CTTCA--ATAATGTGAAAGAAAAAGTCTAGAAAAACCC-TCCAAGATCTTCTCATAGTTT 1822

VvMYBPA2 AGTGCATATTGTGTCAAAGCCTAATTTTAAAAAATTGGACCCAACATCAAGAC---CCAA 1823

* ** *** **** ** * ** **** **** *** *

VvMYBPA1 AAAAGGGGGAGATGAGGGTGGGAGTTCTTGTGCTCAGCAGAGCATCAGCTTCATAGCTTT 1882

VvMYBPA2 AGCACTGGCACATGATGGTTTCTT-----ATCTTCGTTTTTTCTTGA---TTCTAGGAGG 1875

* * ** * **** *** * ** * * * * ***

VvMYBPA1 AGTTTTCCTTTCTGTCCCTTGATTCTCTCTCAC---TTTCTCGTGTCCCAAACGCGGGAA 1939

VvMYBPA2 TGTTTAAGGCACGTTGGTGGGTTTCTCCCAGCCTTATATATGGGATCTATAAATGGGGTT 1935

**** * * * ***** * * * * * * ** ** ***

VvMYBPA1 AGAAAATAAACAAAAGTTGTACTTGTAGAGTGATAAGGGAAAGAAAGGAAAGAGAGAGAT 1999

VvMYBPA2 C------------CAATCGTCCATACACAG--CTTGGTTCAGAGAGAGAGAGAGAGAGAC 1981

* * ** * * * ** * * * * ** *********

VvMYBPA1 ------------------ 1999

VvMYBPA2 TTTTTGTAGAGAATATAA 1999

**Fig. S3** **Sequence alignment and MBS prediction of the *VvMYBPA1* and *VvMYBPA2* promoters**.

The 2000 bp fragments upstream of ATG for *VvMYBPA1* and *VvMYBPA2* were selected as their promoters. Sequence alignment was performed at <https://www.ebi.ac.uk/Tools/msa/clustalw2/>. MBS and MRE, which are highlighted in red and yellow, respectively, were predicted at <http://bioinformatics.psb.ugent.be/webtools/plantcare/>


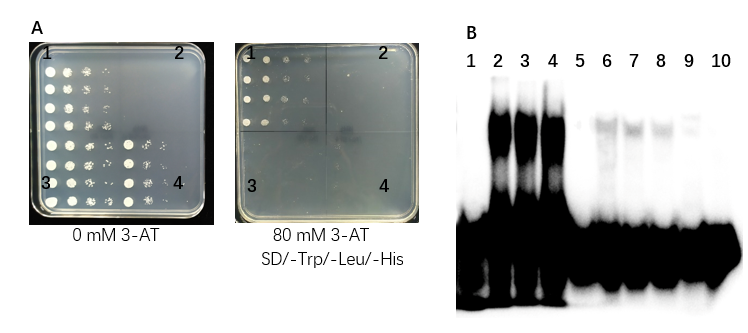


**Fig. S4** Characterization of the binding of the VvMYB14 protein to the MRE (MYB recognition element) of the *VvMYBPA1* promoter.

(A) Y1H assay. 1) p53::pHis2+pGADT7-Rec2-p53, Rec2-p53 and the p53 promoter, whose interactions have been confirmed, acted as positive controls; 2) pHis2+pGADT7; 3) MRE::pHIS2+pGADT7; 4) MRE::pHIS2+pGADT7-MYB14. In panels 1–4 of the photos corresponding to 0 and 80-mM 3-AT, the yeast cells were diluted 1-, 10-, 100-, and 1000-fold, respectively, from left to right. 3-AT was used as a screening marker.

(B) Interaction of the MYB14-His protein with the labeled DNA probes containing MRE elements within the MYBPA1 promoter in an EMSA. The interaction of MYB14-His protein with the labeled probes containing MBS elements were used as positive controls. 1) labeled probes containing MBS elements; 2-4) labeled probes containing MBS elements + MYB14-His proteins; 5) labeled probes containing MRE elements; 6-8) labeled probes containing MRE elements + MYB14-His proteins; 9) competitor probe (5×)+ MYB14-His proteins; 10) competitor probe (10×)+ MYB14-His proteins.

When 80 mM 3-AT was applied, the growth of yeast cells containing MBS::pHIS2+pGADT7 was inhibited; those containing MBS::pHIS2+pGADT7-MYB14 showed very weak growth. Additionally, EMSA indicated weak binding between the VvMYB14 protein and the VvMYBPA1 promoter fragment containing MRE, and binding was inhibited by the application of unlabeled MRE. Therefore, VvMYB14 weakly bound to the MRE element of the *VvMYBPA1* promoter.

GTTTTTTTTTAAGTATATGTTTCTAAGGAGATAAATTTGAAATTTAAATAAATAAACTAGGGATGAATTTAATTATTATTATTTATTTTATTTTATTTCTTCAACTCAAAACAAGTTTAGGTATTATCTTGTCTCATCTCACTTTGTCTTACCTTAATTCTATATAAAATTAATTTAATTTAGTTTTTATTTTCTTATTTTAAAATATAATAATAATAATAATAAAAATATTTTTCAATAGTATATGTTTTTAAAAAACTTATAATATTTTAATTATTTATAAAATATATTTATTTTAATCTAATTAATATTTTTAAAAGGAAATTTAATTTTTTTTAAAAAAAAAGTTAAACGAAAGGAAAAGGATAGGTATGAGAATGCACATATTCATTCCACTTGCATTCCGCCCCATTTATTTATTTTTAATGAAATAAAGATAATAATTATTTTGAATAAACAGATGATTAGGATAGAGGTGACGCATCCAAAACTGCTCCATTGTTATCCCTATTAATTTTAGAAAAAACAAAAACCTATAAATATATAAATAAATAAAACTTCATAAATATCTTATTCCAATCAATAGAATGATAACTCCTCTCTATTGAATCCTCTCTTATACCTCAGTGGGCATTTTCTCAAAAAAAAAGGTTCATTTATATGACTTATCTTCTTTTCCCTTCTCTTCTTATCCTCTTTAAACTTCTAGCATCTTCTTTTAGGATGTAAATTAATTATAATCATGAGAGAATATATATATATATATATATAGTGGTAATCCAAATAATAAAGTGTCCTTATCCTACTTGAGTAACATTCAAGGCATTGCCTGGATTTTAAAATGACTCACCCCATCCAACCACAAGTCAGCCGCCACAAATAATGCACATGCATGGACCAGCCCCAACTTGTCTGACGTCTACCTTTATAGAACATTCTTCGAACTAACCAGGAACCTCATCCGAAACCTCCTCTTTCCATATATGTGTATATATACACACCACAACCTCATGTTTGAAGCACAGAGGTCCACTTACTCACTTCTCTTTACCCTCGTCTCTCTTTCTCAACTTGTTTCGACTTTCAACCCTAGACTATTTCTCACTGCTCATCTCCAATC

**Fig. S5 The 1000 bp fragments upstream of ATG for VvERF104**.

MBS (CAACAG) and MRE (AACCTAA) were not found in this fragment. ERF elements are highlighted in red.

**Fig. S6 Growth increases of WT, *MYB14*-overexpressing and MYB14-suppressing grape calluses**.

The growth increase (fold) was calculated by comparing the fresh weight of grape calluses at 0 and 35 days after subculture. The values indicated by the same lowercase letters are not significant at P < 0.05 on the basis of Duncan’s multiple range test.

**Fig. S7** Leaf number of the WT and VvMYB14-suppressing grapevines


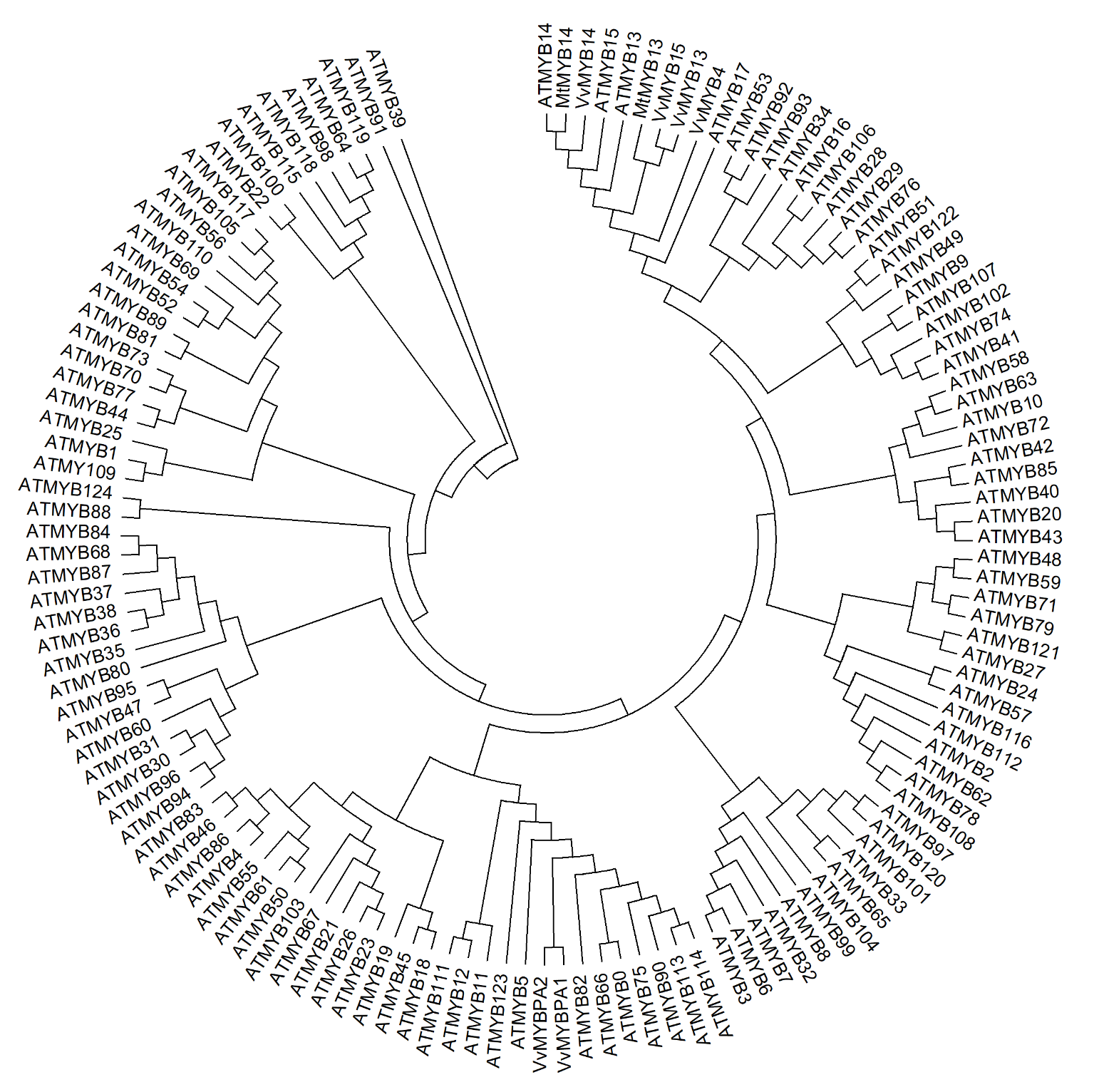


**Fig. S8 Phylogenetic tree of the selected VvMYBs, MtMYB14 and AtMYBs**. The tree was constructed with MEGA 4.1 software.

MtMYB14 MVRAPCCEKKGLKKGPWTLEEDEILTSYINKHGHSNWRALPKHAGLLRCGKSCRLRWINY 60

VvMYB14 MGRAPCCEKMGLKKGPWTPEEDQILVNYIHLYGHGNWRALPKQAGLLRCGKSCRLRWTNY 60

* ******* ******** ***:**..**: :**.*******:************** **

D R

ELXXKXXXLXXXXXXLXXXR

MtMYB14 LKPDIKRGNFTNEEEETIIKMHESLGNRWSAIAAKLPGRTDNEIKNVWHTHLKKRLLNTN 120

VvMYB14 LRPDIKRGNFTSEEEETIIELHERLGNRWSAIAAKLPGRTDNEIKNVWHTHLKKRLKHNH 120

*:*********.*******::** ******************************** :.:

MtMYB14 NNQPNSNTKKRVSKQKIKRSDSNSSTLTTASNCTFS--------------------SDFS 160

VvMYB14 ATPPPKRHSLDASQVEKQQNPINSATNSRSESLGYGPVLSPQPSFSDISSAATTTTTTTT 180

. * .. . .*: : ::. **:* : :.. :. : :

MtMYB14 SQEKNLDNSIICEDSLVTMPEIDESFWSETVIDDEISSTMPSNSMTVSNDLPDQQCIFNN 220

VvMYB14 ATMSDITTPCIKVDSPEDFPEMDENFWSEVLSSNKSGAAG---DLPGAASGPQLQFPFSP 237

: .:: . * ** :**:**.****.: .:: .:: .: : . *: * *.

MtMYB14 SVENFQNPFDNDDDGMDFWYDVFIKSGESTELPEF 255

VvMYB14 RAVIGSSPYSTYDMDMEFWYNIFTRSGELHELSEI 272

. ..*:.. * .*:***::* :*** ** *:

**Fig. S9** **Sequence alignment of VvMYB14 and MtMYB14**.

Sequence alignment was performed online at <https://www.ebi.ac.uk/Tools/msa/clustalo/>. The [D/E]Lx2[R/K]x3Lx6Lx3R amino acid motif was indicated with red box.

Sequence identity=49.45%
